# The Living Space: Psychological Well-Being and Mental Health in Response to Interiors Presented in Virtual Reality

**DOI:** 10.3390/ijerph182312510

**Published:** 2021-11-27

**Authors:** Nour Tawil, Izabela Maria Sztuka, Kira Pohlmann, Sonja Sudimac, Simone Kühn

**Affiliations:** 1 Lise Meitner Group for Environmental Neuroscience, Max Planck Institute for Human Development, 14195 Berlin, Germany; sztuka@mpib-berlin.mpg.de (I.M.S.); sudimac@mpib-berlin.mpg.de (S.S.); 2 Clinic and Policlinic for Psychiatry and Psychotherapy, University Medical Center Hamburg-Eppendorf, 20246 Hamburg, Germany; kira.pohlmann@studium.uni-hamburg.de

**Keywords:** indoor architecture, interiors, contours, affect, behavior, cognition, spatial experience, virtual reality, well-being, mental health

## Abstract

There has been a recent interest in how architecture affects mental health and psychological well-being, motivated by the fact that we spend the majority of our waking time inside and interacting with built environments. Some studies have investigated the psychological responses to indoor design parameters; for instance, contours, and proposed that curved interiors, when compared to angular ones, were aesthetically preferred and induced higher positive emotions. The present study aimed to systematically examine this hypothesis and further explore the impact of contrasting contours on affect, behavior, and cognition. We exposed 42 participants to four well-matched indoor living rooms under a free-exploration photorealistic virtual reality paradigm. We included style as an explorative second-level variable. Out of the 33 outcome variables measured, and after correcting for false discoveries, only two eventually confirmed differences in the contours analysis, in favor of angular rooms. Analysis of style primarily validated the contrast of our stimulus set, and showed significance in one other dependent variable. Results of additional analysis using the Bayesian framework were in line with those of the frequentist approach. The present results provide evidence against the hypothesis that curvature is preferred, suggesting that the psychological response to contours in a close-to-reality architectural setting could be more complex. This study, therefore, helps to communicate a more complete scientific view on the experience of interior spaces and proposes directions for necessary future research.

## 1. Introduction

Built (man-made) environments have become fundamental components of human existence. For the majority of our waking time, we navigate and interact with architectural environments while we live, connect, learn, work, and recreate. The spaces encountered in daily life vary in their physical and aesthetic properties, and may have an influence on affect, behavior, and cognition, and eventually impact mental health and psychological well-being [1,2]. These effects are likely the outcome of an interaction between the physical properties of the perceived space on the one hand, and the perceiver’s characteristics and the meaning they create on the other [3,4,5].

When accounting for the considerable time spent inside buildings, two-thirds of which is in dwellings [6], the glaring gap in linking variations in physical features of architecture to psychological states is surprising [7,8]. It has been previously suggested that this can be attributed to methodological and disciplinary incongruences between architecture and psychology [7,8,9]. Architectural research connecting the human response to design relies on philosophical constructs, whereas traditional psychological research investigating the human–environment relationship relies on observation and subjective measures [9,10]. A better understanding of the human–environment interaction could contribute to informing design strategies in ways to optimize psychological well-being and mental health [11]. Although the discussion has been initiated, a commonly accepted methodology across disciplines is still lacking [12].

A domain in which first successful attempts have been made to link architectural features to psychological responses in human beings concerns contours. We refer to contours here to describe the “edge or line that defines or bounds a shape or an object” [13]. The interest in the response to contours derived from empirical studies in various disciplines such as arts, aesthetics, visual cognition, and (social) psychology among others, which have reported differences in perception. Early studies from the first quarter of the 20th century have found that straight lines were associated with unpleasant “feeling tones” that denote strong motor expression (e.g., agitating, hard, furious, and serious), whereas curved ones were associated with adjectives indicating relatively more pleasantness and less movement (e.g., gentle, quiet, and lazy) [14,15]. Subsequent studies have investigated the hypothesis that curved/rounded/curvilinear conditions are more appealing to humans than angular/edgy/rectilinear ones. This hypothesis has been shown to be correct using different types of visual stimuli including lines [14,15,16,17,18], font types [19,20], geometric shapes and simple forms [21,22,23,24], irregular shapes and meaningless patterns [3,4,16,22,23,24,25,26,27,28,29,30], images of familiar objects [3,26,31,32,33,34,35,36], sketches of familiar objects [33,37], in addition to sketches and images of designed products [38,39]. Different studies have found the effect to be present across species humans and apes [35], cultures—Western vs. non-Western [14,16,19,24,29,35,38,39,40], and ages—toddlers [27] and infants [18,23]. However, the source of this preference is still under debate. Some researchers proposed that angularity conveys threat, suggesting that the preference reflects adaptive behavior [31,32]. Other research has attributed the observed effect to higher cognitive processes and susceptibility to the influence of semantic meaning and perceptual qualities that are not strictly limited to contour [35]. Conversely, additional studies proposed a “curvature effect” that was not linked to a negative response to angularity for what it affords but rather caused by intrinsic characteristics of the curved stimuli [29], with preference modulated by positive valence [34]. Moreover, other studies have investigated additional variables beyond simple curves and angles. Those included both properties of the stimuli—e.g., complexity [22,24,28,29,30,38], symmetry [24,36], balance [22,24], novelty/innovativeness [38], meaningfulness [26,29], typicality [38,39], familiarity [4,33], as well as individual differences of the perceivers—e.g., sex [3,27,30], expertise in art/design [3,4,24,33,38], academic degree [33], personality traits [3,22,33], cognitive styles [26], and neurological disorders such as autism [4,21,30], in an attempt to understand whether they affect or modulate contour perception. Different outcome measures have been used in previous studies, including forced-choice response [29,31,32], rating/visual analogue scales [4,16,19,20,21,22,24,29,30,33,37,39], and selection procedures [26], in addition to more implicit measures, such as association [14,17,20,25,28] and approach-avoidance tasks [3,16,28,36], reaction and/or viewing time [18,22,26,27], and observed postural behavior [21]. With regard to contours in the indoor environment, similar effects were proposed by the scarce set of studies available until now. Spaces with curvilinear/curved features, in comparison with those with angular/rectilinear ones, were preferred among different ages [41], and induced higher positive emotions such as pleasure [42,43,44,45], relaxation, safety, privacy [46], and a desire to approach [44]. The majority of these studies relied largely on subjective semantic scales, where stimuli were rated according to a limited list of paired opposite adjectives to depict emotional responses (i.e., valence, arousal, approach-avoidance, and some spatial properties). It is worth noting that the stimuli used, for the most part, did not reflect realistic environments. More recent research used different approaches and new experimental tools to investigate the architectural experience. The effect of contour on aesthetic judgment and approach-avoidance decisions was examined in one of the very first functional magnetic resonance imaging (fMRI) studies to examine architectural perception [8]. Images of existing real-life indoor environments were presented for three seconds in the scanner, and participants rated each image and used a joystick to indicate whether they would like to enter or exit the environment. Results showed that curvilinear interiors were more likely judged as beautiful, compared to rectilinear ones. Moreover, they were found to activate the medial orbitofrontal cortex—titled as anterior cingulate cortex (ACC) in the publication exclusively, which has previously been related to positive valence and pleasantness [47]. In contrast with previous fMRI evidence from studies investigating familiar objects [31], no amygdala activation for rectilinear spaces was found. Consequently, given the amygdala’s role in processing information related to fear and arousal [48], the results did not confirm the hypothesis of the threat effect evoked by angularity. Additionally, unlike what was hypothesized, contour did not affect approach-avoidance decisions. The stimulus set was partially tested in more recent studies that examined individual differences. Eight images were presented to experts and laypersons [49], and 80 to quasi-experts, individuals with autism spectrum condition (ASC), and a matched neurotypical group [4]. Results were not consistent across the different studies, with the latest one finding a preference for rectilinear spaces within all three groups. Two major setbacks may have caused the inconsistencies between the reported results. The first concerns the use of 2D images (static stimuli) to represent realistic environments and investigate a real-life experience [50], and the second relates to the fact that the stimuli were not well-matched. Creating controlled testing environments in which separate architectural design features can be altered and tested each at a time represents, in fact, one of the main challenges in quantifying the impact of design on human experience [7]. With the recent technological advancements in virtual reality (VR) and computer-aided design (CAD) software, it is now possible to develop experimental settings that can replicate the experience of a real environment under controlled conditions [51,52], while evoking similar user responses [53,54]. Combining human monitoring techniques with advanced VR environments can enable the acquisition of objective evidence for evaluating the human response to indoor design [9,52,55]. For example, one study investigated different interior form features using VR combined with electroencephalogram (EEG), during active exploration of empty white-colored virtual environments [10]. Results showed higher pleasure and arousal ratings and increased theta activity in the ACC when exploring curved geometries, as opposed to more linear ones. However, source localization of the EEG signal in the brain is a complex task with forward and inverse problems, calling the exact location of the source ACC into question. Another example study examined neurophysiological and behavioral responses during the appreciation of virtual environments, using EEG and explicit ratings of novelty, familiarity, comfort, pleasantness, and arousal [56,57]. Despite the fact that the two virtual rooms used in the studies represented contrasting contours (i.e., the “modern design” room had angular furniture, and the “cutting edge design” room displayed furniture with rounded edges), the researchers rather focused on style in their categorization of the stimuli. Whereas the interest of the study was not in finding a preferred environment, but rather to explore the relationship among each of the perceptual dimensions and correlate them with brain activity, modern and cutting edge environments were perceived, respectively, as more familiar and more novel, but no differences in ratings of pleasantness, arousal and comfort were reported. Taken as a whole, the evidence for curvature preference, although seemingly robust with abstract shapes and lines, is yet far from being confirmed in the context of indoor architecture, and requires further thorough investigations.

Another line of research exploring the response to built environments has investigated the restorative properties of indoor spaces. The attention restoration theory (ART) proposes that natural environments, filled with “soft fascinations”, could restore cognitive capacity, reduce mental fatigue, and increase focus and attention [58]. Being in restorative environments could, therefore, change negative states to positive ones. Building on the biophilia hypothesis, which suggests that humans have an innate connection with nature [59], a framework for biophilic design has emerged [60]. By bringing elements of nature into living spaces (directly or indirectly), positive effects might be initiated. Studies investigating biophilic interventions in virtual indoor environments have found a stress reduction and restorative effect [61,62].

Within the scope of the present study, we aimed to systematically examine the influence of contours (angular versus curved) in virtual indoor architectural settings on affect, behavior, and cognition. Given the significant time urban dwellers spend in the home environment, which has considerably increased since the COVID-19 outbreak in March 2020, we selected the residential space as the context of our present investigation. Interiors are considered a major part of architecture, more than ever in the revolutionary works of modernist architects who regarded interior spaces as the essence (e.g., Bruno Zevi, Hans Scharoun), highlighted the importance of furniture, and influenced modern furniture design (e.g., Alvar Aalto, Marcel Breuer). Furthermore, we wanted to account for the evidence that architectural style and layout influence the response to form [44], and to architecture per se, knowing that results of studies on the perception and evaluation of style are inconsistent [63,64]. Hence, we included style as an explorative second-level variable. Previous studies investigating aesthetic styles have used classifications such as modern/contemporary vs. classical [65,66,67] vs. traditional [68], among others. We opted for “modern vs. classic” for the interdisciplinary potential of the dichotomy. We refer to “classic” to denote the variant styles of the traditional abacus of architecture, up to the beginning of the 20th century [69]. “Modern”, on the other hand, refers to the architecture of both 20th and 21st centuries, starting from modernism and the stream of styles it inspired by completely breaking with the past [70].

As we aimed to delve deeper beyond the mere investigation of pleasantness, beauty, and arousal, our behavioral measures covered a larger set of affective and psychological dimensions, for a better overview of spatial perception. To inspect the impact of contour on cognition and restorativeness, we included a measure of perceived restored attention, building on the attention restoration theory (ART) [58], and a mental arithmetic task from the Trier social stress test [71], previously used in environmental VR studies [72]. We present here a new paradigm that allows the collection of both explicit and implicit measures of the human response to indoor environments while allowing for a close-to-reality experience. To the best of our knowledge, none of the previous studies have explored high-quality photorealistic, yet well-matched virtual stimuli representing contour contrasting conditions within a free-exploration setting, while controlling for style. Extending on the findings of the scarce studies inspecting contours in the architectural context [8,10,44,46,49,73] and the seemingly robust scientific and empirical evidence supporting curvature preference in other domains (references above), we expected curved conditions to positively impact the self-reported emotional and spatial experience, and to improve cognitive performance as well as the self-reported feeling of restorativeness.

## 2. Materials and Methods

### 2.1. Participants

A sample size estimation using G*Power—version 3.1.9.7 (Dusseldorf University, Dusseldorf, Germany), resulted in the need for 36 participants to enable medium effect size. Due to the high potentiality of technical errors, and the increasing rate of cancelled sessions as a result of the COVID-19 pandemic, the recruitment process was kept open until reaching *N* = 36 individuals who provided usable complete sets of behavioral VR data. Eventually, 48 healthy adults were enrolled, aged between 18 and 40 years, with no severe visual impairments. Further inclusion criteria included fluency in German language and absence of diagnosed mental or neurodegenerative disorder or cognitive impairment. Subjects were recruited through the Castellum Database of the Max Planck Institute for Human Development in Berlin (MPIB) and an online platform (https://www.ebay-kleinanzeigen.de/) and were compensated with 10 euros/h. All participants signed the consent form before the experiment.

### 2.2. Stimulus

**Figure 1:**
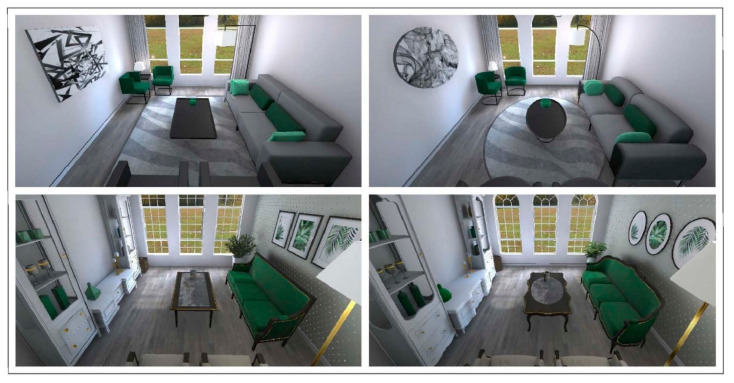
Virtual 3D environments, created for the study. Upper images display the modern style, and the bottom ones the classic style. **Top left:** angular modern (AM). **Top right:** curved modern (CM). **Bottom left:** angular classic (CA). **Bottom right:** curved classic (CC). Images were taken from the Unity project with a perspective that does not represent a human eye view, to show maximum coverage of the room.

Two pairs of living rooms were created for the purpose of the study (Figure 1). Rooms of each pair were identical in their design, except that one had angular window openings, furniture, fixtures, accessories, patterns, and other specific details, while the other had curved counterparts. The main contrast between the pairs was style (classified under: modern vs. classic), with some differences in layout, furniture components, and materials, which were seen necessary to reflect the style. “Classic rooms” included features from the neo-classicism period (e.g., “ornamental” furniture of Louis XV and VI style; wallcovering; detailed door and windows; more objects in the room), while “modern rooms” followed the “less is more” principle (e.g., Ludwig Mies van der Rohe) in a minimal style (e.g., less detailed furniture, door and windows; less objects in the room). Moreover, the classic pair included elements of biophilic design (e.g., wood furniture, plants, images of plants, more surfaces with green color). The main challenge was to design objects/elements that reflect well-proportioned, yet matching counterparts in both contour versions, without causing a change to style or familiarity. Hence, furniture design was inspired from common pieces that exist in both contour versions, although changing contours or proportions of famous designer pieces was completely avoided. In order to control for additional confounding factors, rooms’ boundaries, ceiling, floor, door and windows locations, main seating positions, main light, and primary color (green) were kept identical between the pairs, in addition to the outdoor window view portraying a natural environment.

**Figure 2:**
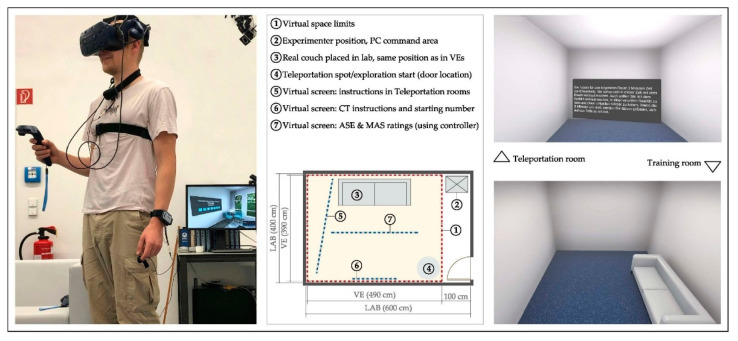
Virtual reality (VR) laboratory set up. **Left side:** VR setup in pilot session, participant about to start responding to rating scales. The physical couch is shown on the right side of the participant. **Middle:** general layout showing the virtual space in relation to the actual laboratory conditions, experimenter position, physical couch position, starting point of each exploration task, and virtual screens that appear successively during the paradigm. **Top right:** teleportation room, presented at the start of the VR session, and in between each of the rooms. **Bottom right:** training room, simulating the laboratory appearance.

The size of the virtual room was similar to the MPIB VR lab space dimensions and fixed accordingly to (L × W × H = 4.9 × 3.9 × 3 m) so that free movement was possible during participants’ exploration. Three-dimensional models of all the objects and details of the rooms were created using 3Ds Max—version Theseus, 2020 (Autodesk Inc., Mill Valley, CA, USA), and the paradigm with all the tasks was implemented using the gaming software Unity—version 2019.2.1f1, 64-bit (Unity Technologies, San Francisco, CA, USA). The rooms were rendered in real-time during the experiment, using Unity High Definition Render Pipeline (HDRP, version 6.9.1) and were displayed with Steam VR (Valve Corporation, Bellevue, WA, USA)—multiple updates during experiment, no standing version to report, through an HTC Vive Pro headset (HTC corporation, New Taipei, Taiwan), connected to a wireless adapter to allow for unobstructed movement. In order to increase immersion, a real physical large couch was included in the set-up, positioned at the same location as in the virtual rooms. Participants could use it within their exploration time, and were asked to sit on it to perform the cognitive tasks (Figure 2).

Additionally, a virtual training room was created, simulating the lab appearance (Figure 2) including the physical couch, with one version having angular edges (for the couch, lighting fixture, and door accessories), while the other had curved counterparts.

As the study comprised a within-subject design, all participants were tested under all conditions in randomized order, always starting with the training room. Participants with odd ID numbers were first exposed to the training room with curved features, while those with even IDs were assigned to the one with angular ones. Using counterbalancing through a Latin square design, and after eliminating sequences where rooms of the same pair would have been shown successively, four groups were identified, to which participants were randomly assigned: Group A (AM, AC, CM, CC); Group B (AC, CM, CC, AM); Group C (CM, CC, AM, AC); and Group D (CC, AM, AC, CM), where AM is “angular modern”, CM is “curved modern”, AC is “angular classic”, and CC is “curved classic”.

### 2.3. Measures

The main part of the experimental paradigm consisted of the VR session. In each room, participants started exploring the virtual space for 3 min, followed by a 2-min cognitive task in a sitting position, and responded to a set of questions. Multiple questionnaires were administered before and after the VR session (we mention below only those included within the present analyses).

#### 2.3.1. Questionnaires

The in-VR questionnaires included two sections assessing respectively the affective and spatial experience (ASE), and momentary affective state (MAS). ASE consisted of 20 items related to the subjective perception of emotional and spatial dimensions. Participants provided self-reports on 20 bipolar (−5 = “describes strongly”, 0 = “neutral”, 5 = “describes strongly”) dimensions using 11-point numeric scales, tagged by two opposite descriptive adjectives on each of the sides. Dimensions encompassed valence, arousal, and dominance, but also covered other spatial aspects (e.g., organization, spatiality, naturalness), and were retrieved from previous studies [7,8,56,74], with some additions that were found to be relevant to the study (Table S1). All anchor adjectives were translated to German, for the purpose of this experiment. MAS was assessed using 11-point intensity rating scales for 11 dimensions (original German version used in previous studies [75]). The dimensions assess different domains: emotional feelings, bodily sensation, valence and arousal, and cognitive and motivational states (Table S2). The first 6 scales were unipolar (0 = “little”, 5 = “neutral”, 10 = “very”), followed by 5 bipolar scales tagged by one to four descriptive adjectives as anchors (−5 = “describes strongly”, 0 = ”neutral”, 5 = “describes strongly”). A pre-measure was also collected before the VR session to control for the baseline affective state. In sum, participants responded to 31 dimensions, in-VR, after exposure to each of the rooms, with a total of 155 questions (including the training room).

As part of the post-VR PC-based questionnaire, subjects reported on more aspects of the virtual, spatial, and cognitive experience. Perceived restorativeness (PR) was measured using an adapted 12-item German version of the Perceived Restorativeness Scale (PRS) [76], under four categories: fascination, being away, coherence, and scope (Table S3). Each item was rated on a five-point Likert scale from 0 (not at all) to 4 (completely). 

#### 2.3.2. Cognitive Task (CT)

Cognitive performance was evaluated using the results of an in-VR two-minute skip counting task [72]. After exploring each of the simulated conditions, participants were asked to keep subtracting 13 from a starting 4-digit number that was shown on a virtual screen and to pronounce the intermediate results out loud. When participants made mistakes they were prompted to start anew from the same starting number. The sequence of numbers was the same for all participants, and answers were collected manually by experimenters. Individual scores were calculated by dividing the total number of correct answers (in all attempts) by the number of attempts.

#### 2.3.3. Additional Measures

To evaluate the overall VR experience, and control for specific undesired effects, cyber-sickness was measured using an adapted German version of the Simulation Sickness Questionnaire (SSQ) [77], administered both pre and post-VR sessions. SSQ consists of 16 items based on a four-point Likert scale ranging from 0 (symptom not existent) to 3 (very severe symptom), which can be computed into three representative subscores: Nausea-related (N), Oculomotor-related (O), Disorientation-related (D), in addition to a Total Score (TS) representing the overall severity of cybersickness experienced by participants. Moreover, presence was assessed using the iGroup Presence Questionnaire (IPQ) [78]—adapted from the German version available online (www.igroup.org, Accessed on 15 September 2020) administered post-VR. IPQ contains 14 items rated on a five-point Likert scale (1–5) tagged with different anchors, according to the four sub-scales that measure different components of presence: General Presence (GP), Spatial Presence (SP), Involvement (INV), and Experienced Realism (REAL). Both SSQ and IPQ were administered right after the VR session, as part of the post-VR questionnaire. Additionally, we collected information related to demographics and other individual differences that are beyond the scope of the present analyses.

### 2.4. Procedure

**Figure 3:**
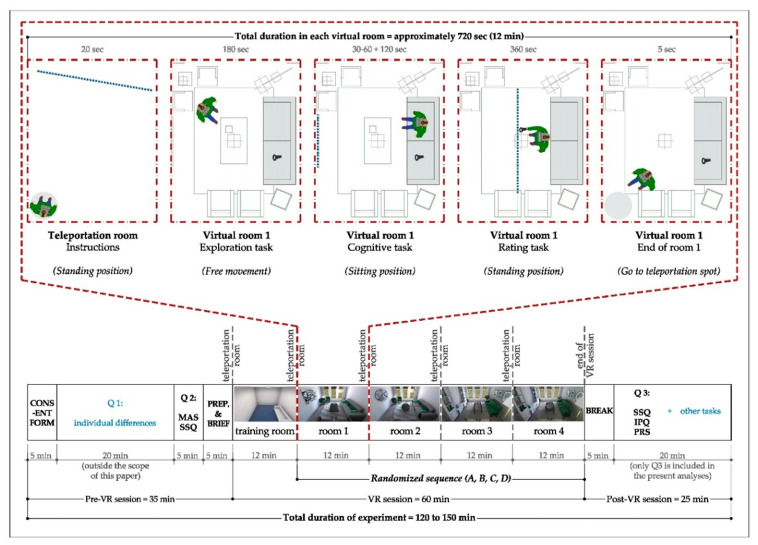
Details of the experimental paradigm. **Bottom:** The bar shows all phases of the experiment (pre-VR, immersive, and post-VR sessions) along with the respective approximate duration. The mentioned durations are based on the average of the time spent by different participants. **Up:** The upper layouts display the sequence of events denoting tasks and instructions in each of the virtual environments. Room AM (angular modern) is displayed in a top view layout as an example. The average duration in each virtual room was 12 min (with an approximate total of 60 min). Some events were fixed and had a predefined time (e.g., teleportation room, exploration tasks, cognitive task), while others depended on the participant’s speed (e.g., reading cognitive task instructions and readiness to start, rating tasks, moving back to teleportation spot). Q1 and tasks in the last section mentioned as “other tasks” are excluded from the present analyses. Note: SSQ = simulation sickness questionnaire, IPQ = IGroup presence questionnaire and PRS = perceived restorativeness scale.

All experimental sessions were conducted in the VR lab at the MPIB (November to December 2020), in compliance with the institute’s COVID-19 regulations for lab hygiene. The experiment was composed of three parts: (1) pre-VR questionnaires and preparation; (2) immersive session; and, (3) post-VR questionnaires and tasks. Participants received the consent form via email before the day of the experiment. They were encouraged to read and sign the form, and to fill in the pre-VR questionnaire before coming to the lab, otherwise, those were completed on the day of the experiment. Upon arrival, participants were presented with an introduction to the study, filled a PC-based questionnaire to collect baseline measures for the affective state and simulation sickness symptoms, and performed a short training session on the cognitive task. Next, they were prepared for the VR session. Details were described thoroughly, the head-mounted display (HMD) was put on with the help of the research assistance staff, and subjects were guided to stand in the teleportation areas next to the room door. The VR session started with an empty teleportation room showing instructions for 20 s, followed by the training room, for familiarization with all in-VR tasks. Each room was simulated for 3 min of free exploration, and participants were encouraged to explore as they needed to, until they felt they could later recognize the room from a photo. At the end of the exploration time, a message was shown at eye level with a message to sit on the couch. Instructions for the cognitive task were displayed on a screen at the wall facing the couch, and when participants confirmed readiness, the starting number was shown. Answers were manually written down by the experimenter, who prompted the participant to “restart” after a wrong number was named, until a “stop” sign was shown at the end of the 2 min. Later, a screen appeared in the middle of the room with instructions on how to answer the questionnaire using the controller. Once all questions were answered, participants were asked to leave the controller on the couch and go to the teleportation spot at the door. The sequence of events and tasks is displayed in Figure 3 (upper side). The process was repeated for all rooms, with the teleportation instruction room presented for 20 s in between. At the end of the immersive session, a sign was shown at eye level indicating “the end”, HMD was dismantled, and participants took a break. The third part of the experiment included the PC-based questionnaire in addition to further tasks that were not used for the present data analysis. Details of the experimental paradigm are displayed in Figure 3.

### 2.5. Data Analysis

We preregistered our research plan, which can be retrieved from (https://aspredicted.org/vp93z.pdf, Accessed on 23 February 2021). During data preprocessing, sessions with technical software/hardware errors, and those where participants requested breaks or showed severe symptoms of simulation sickness were excluded. Out of the 48 participants enrolled, a range of 36–40 (85.36% born in Germany; ASE and MAS: F = 25, M = 16; CT: F = 27, M = 15; PRS: F = 23, M= 13) were included in the analyses (Table S4).

Self-reports assessed with questionnaires (MAS, ASE, PRS) in addition to the cognitive task scores were analyzed using paired samples two-tailed *t*-tests to examine differences in contour (angular vs. curved) and style (modern vs. classic). When the normality assumption was not met, instead of paired sample *t*-tests, the Wilcoxon signed-rank test was used. Statistical tests were performed separately for each of the dimensions of the ASE and MAS. For ease of reference, we will be referring to rooms according to their condition in the following parts of the paper (e.g., angular rooms, classic rooms, etc.).

Considering that we collected 33 separate outcome variables, and conducted two different tests with each (angular vs. curved, modern vs. classic), eventually we had to conduct 66 frequentist statistical tests. When performing multiple statistical tests, one should take into account that setting the alpha value to 0.05 will result in 5/100 significant results purely by chance, in our case 3.3 significant tests. According to the Bonferroni correction method, which strongly controls for family-wise error rate, the critical value for each comparison is the type I error rate divided by the number of comparisons: α/k = 0.05/66 = 0.00076. However, we also checked for the false discovery rate (FDR) correction, as the Bonferroni correction has been considered overly conservative [79]. FDR correction controls for the proportion of “discoveries” (significant results) that are false positives.

To examine if the observed non-significant results in the frequentist approach represent an absence of the predicted relation between room contour and dependent variables measuring mood and cognition, we examined the amount of evidence in favor of the null hypothesis using the Bayesian framework [80]. The BF_01_ in the Bayesian framework indicates how much more likely it is that the data occur given the null hypothesis.

The analyses within the frequentist approach were conducted using R Studio—v1.4 Tiger Daylily (RStudio, Boston, MA, USA), and Bayesian analyses using JASP—version 0.14.1.0 (University of Amsterdam, Amsterdam, The Netherlands).

## 3. Results

### 3.1. Behavioral Measures

#### 3.1.1. Affective and Spatial Experience (ASE)

The paired-samples *t*-test revealed that participants rated angular rooms higher compared to curved rooms on dimensions novelty (*t*(40) = 3.95, *p* < 0.001), order (*t*(40) = 6.20, *p* < 0.001) and symmetry (*t*(40) = 2.13, *p* = 0.039), whereas curved rooms were rated as more exciting (*Z* = 2.01, *p* = 0.044) and harmonious than angular rooms (*t*(40) = −2.39, *p* = 0.022). 

Regarding the room style, modern rooms were perceived as more novel (*Z* = 5.31, *p* < 0.001), more simple (*t*(40) = 6.26, *p* < 0.001), more ordered (*t*(40) = 2.78, *p* = 0.008) and more spacious (*Z* = 2.49, *p* = 0.013) compared to classic rooms, while the latter were rated as warmer (*t*(40) = −3.23, *p* = 0.002) and more enclosed (*Z* = −1.45, *p* = 0.014) than modern rooms.

**Figure 4:**
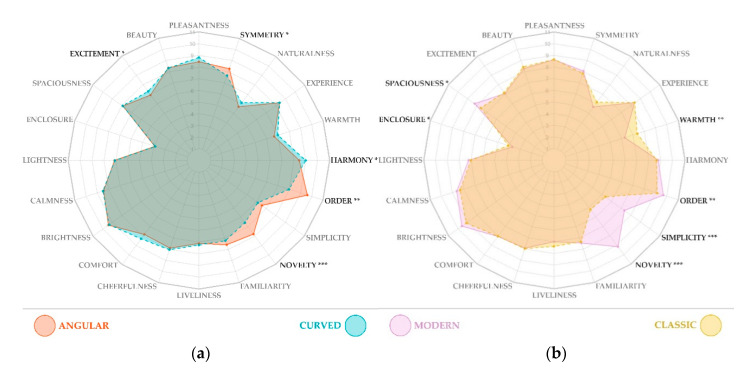
Display of participants’ responses to the bipolar dimensions of the affective and spatial experience questionnaire. The scales were converted from (−5, 0, 5) to (1–11) for analysis and display purposes. Individual scores were calculated based on averaging responses to every two rooms presenting the same condition, and the charts’ scores represent means on each of the dimensions. Plot (**a**) displays results for contour conditions (angular vs. curved), and plot (**b**) shows results of style conditions (modern vs. curved). Significant dimensions are marked with asterisks (*** for *p* < 0.001, ** for *p* < 0.01, and * for *p* < 0.05) and are written in black color for ease of reference. These graphics were created in R Studio, using package fmsb (Minato Nakazawa, 2021, Available on https://cran.r-project.org/web/packages/fmsb/index.html).

We found no statistically significant difference in any of the dimensions: pleasantness, beauty, lightness, calmness, brightness, comfort, cheerfulness, liveliness, familiarity, experience, and naturalness neither for contour nor style comparisons. Participants’ responses on the affective and spatial dimensions are illustrated in Figure 4.

#### 3.1.2. Momentary Affective State (MAS)

**Figure 5:**
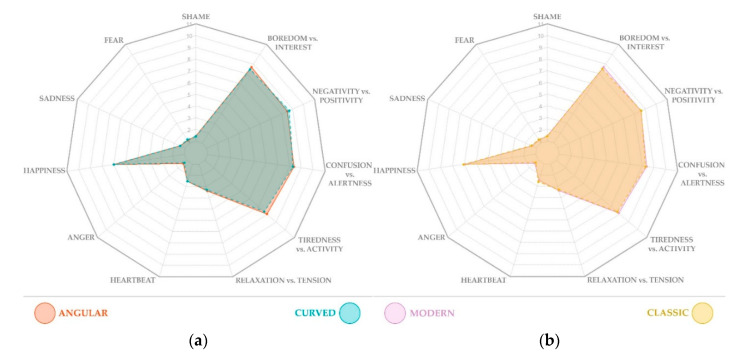
Display of participants’ responses to the momentary affect questionnaire. Emotional feelings and bodily sensation were rated on unipolar scales (0–10), while arousal and valence (tension, activity, positivity) and cognitive (alertness) and motivational (interest) states ratings were presented on bipolar scales (−5, 0, 5). Both scales were converted to (1–11) for analysis and display purposes. Individual scores were calculated based on averaging responses to every two rooms presenting the same condition, and the charts’ scores represent means on each of the dimensions. Plot (**a**) displays results for contour conditions (angular vs. curved), and plot (**b**) shows results of style conditions (modern vs. classic). These graphics were created in R Studio, using package fmsb (Minato Nakazawa, 2021, Available on: https://cran.r-project.org/web/packages/fmsb/index.html).

Contrary to our hypothesis, we did not find any effects of contour on momentary affective states. Moreover, Bayesian factors show that the evidence for no effect ranges from anecdotal evidence for a null result for contour effect on the self-report of being active (BF_01_ = 1.16), to moderate evidence for a null result in the case of self-reported fear (BF_01_ = 8.41). Similarly, there was no effect of style on momentary affective state, and Bayes factors span from anecdotal evidence for the absence of the style effect—on the heartbeat (BF_01_ = 2.00) up to moderate evidence—in the case of alertness (BF_01_ = 5.37). Participants’ responses are shown in Figure 5.

#### 3.1.3. Perceived Restorativeness (PR)

There was no difference in self-reported restored attention after having been immersed in angular compared to curved rooms—*t*(35) = −0.79, *p* = 0.436, nor in modern compared to classic rooms—*t*(35) = −0.94, *p* = 0.352. Bayesian factor indicates that there was anecdotal evidence in favor of an absence of effect of contour (BF_01_ = 2.7) and moderate evidence of absence of style effect on perceived restorativeness (BF_01_ = 3.7).

### 3.2. Cognitive Task (CT)

In line with the behavioral data, we found no effect of contour (*Z* = −0.43, *p* = 0.667) or room style (*Z* = 0.59, *p* = 0.552) on cognitive performance. The evidence in favour of null results is moderate for both contour (BF_01_ = 5.12) and style (BF_01_ = 4.79) effects on cognitive performance.

### 3.3. Virtual Reality (VR) Experience

A paired sample Wilcoxon signed-rank test indicated a significant increase of the overall cybersickness symptoms experienced by participants (*Z* = 4.5423, *p* < 0.001) from pre- (*Mdn* = 7.48, *IQR* = 22.44) to post- measurements (*Mdn* = 26.18, *IQR* = 29.92) (Table S5). This suggests that the total stay in VR increased the simulation sickness symptoms. We calculated a (post-pre) total score and performed additional analyses to control for the effect of simulation sickness on participants’ responses. However, no main effects were found.

IPQ scores were computed for each of the subscales. On a 1–5 scale, mean scores were respectively GP (*M* = 3.93, *SD* = 0.91), SP (*M* = 3.44, *SD* = 0.63), INV (*M* = 3.27, *SD* = 0.98), and REAL (*M* = 2.68, *SD* = 0.47), indicating above medium values for all the subscales, and an acceptable feeling of presence.

### 3.4. Additional Analyses 

Out of the 66 frequentist tests we conducted, we expected 3.3 to be significant by pure chance. However, five returned significance in contour comparison, and six in style (marked with asterisks in Table 1 and Table 2). When applying the Bonferroni method, with the corrected threshold of *p* < 0.00076, four tests survived the correction: novelty and order in contour comparison in favor of angular rooms, in addition to novelty and simplicity in favor of modern rooms when comparing style. However, when applying a less stringent correction method, the FDR correction, warmth remains significant in style comparison with classic rooms perceived as warmer than modern ones.

In line with the frequentist approach findings and the FDR correction, the harmonic mean of the Bayesian factors BF_01_ only indicated strong evidence for the alternative hypothesis (<0.1) in the case of the five aforementioned dimensions in the respective comparisons. All statistical tests and Bayes factors are reported in Table 1 and Table 2.

## 4. Discussion

Within the scope of the present study, we primarily examined the potential psychological response to indoor virtual living rooms with contrasting contour conditions (angular and curved) on affect, behavior, and cognition. Such findings would contribute to understanding the relationship between humans and the built environments they occupy, and would inform the design of therapeutic settings in ways to optimize cognitive functioning, physical and mental health, and well-being. The very few studies that have investigated these conditions in indoor architectural settings have used for that purpose either photos of existing spaces [4,8,49], computer-generated three-dimensional images in color [45] and greyscale [44], sketches and line drawings [46], or schematic virtual environments where the overall form of the room was manipulated [10,73]. Most of these studies reported a preference for, and higher positive emotion in curved/curvilinear conditions as opposed to angular/rectilinear/linear ones (references above), with more recent studies reporting an opposite effect [4]. This may be the result of problems that are prominent to this new field of study [12], among which is a lack of systematic development of a coherent theoretical and experimental framework [57]. We took several measures in an attempt to address some of the methodological shortcomings of previous studies. The first one concerns the nature of the stimuli. For that, we ensured that the virtual environments presented are fully matched in terms of contour contrast, and avoided the possible effects of other confounding variables (e.g., lighting conditions, outside view, room size, floor finish, ceiling height and finish, door location and size, and so on). All these variables were kept identical in all four simulated rooms. Moreover, we included a second level-variable, architectural style, so that we presented to participants a variety that could cover different aesthetic preferences, noting that findings of previous studies were inconsistent with regard to preference. The second shortcoming is related to the lack of real-life architectural experience in previous studies and the predominant use of static stimuli. Therefore, we opted for a VR set-up that stimulates 3D rather than 2D perception, with a free-exploration paradigm and no restrictions on the path; subjects were able to explore the space from different viewing angles, whether standing, sitting, or crouching to see a specific detail. Moreover, we presented high-quality and detailed immersive environments, which were created via high-definition photorealistic instant renderings and post-processing methods (videos of the room can be found on https://drive.google.com/drive/folders/1rIPx0GBHubAsQaWxBWnkY7odOPXn_yiL, Accessed on 19 September 2021). However, we respected the recommended guidelines to reduce VR-induced symptoms and effects by providing high-quality graphics and ensuring that the immersive session did not exceed the recommended maximum duration [81]. Moreover, we familiarized all our participants with the VR system by means of a training session. Third, with regard to outcome measures, we aimed to extend beyond the limited conventional ratings of valence and arousal, criticized by some as not representative of the spatial aesthetic experience [53]. Hence, we included a relatively large set of affective, behavioral, and cognitive measures. Extending from previous evidence on the curvature preference and positive affective effects in both non-architectural and architectural settings, we expected the curved conditions to positively influence momentary affect, emotional and spatial experience, cognitive performance, and perceived restorativeness. 

To our surprise, we did not find relevant positive effects of contour in most of the outcome measures, although the study had a comparably large sample size (e.g., N = 18 [8], 17 [10], 71 × two groups [49]). In fact, the only differences observed between the two contrasting conditions, after correcting for false discoveries, favored angular versions on “novelty” and “order” ratings. This finding stands in contrast to some experimental studies where ratings of pleasantness, attractiveness/beauty, and arousal indicated more positive responses to curved rather angular conditions (e.g., [8,44,46,49]). In particular, we were surprised that in our analysis, differences in self-reports on both “pleasantness” and “beauty” were statistically insignificant. While results indicated a non-significant trend in the predicted direction indicating higher pleasantness ratings for rooms with curvature, the Bayes factor indicated no evidence in this direction. As for beauty, ratings’ means were very similar in both conditions. This is in line with a previous study, where contour had no effect on beauty judgments in laypeople [49]. We also did not find any differences in ratings of arousal dimensions neither in ASE nor in MAS (e.g., excitement, liveliness, calmness, interest, tension, heartbeat, alertness, activity). In terms of momentary affect in general, our results mainly indicated no evidence for the threat hypothesis, as ratings on “fear” were at the lower extreme in both conditions. As a matter of fact, all scores on negative affect were considerably low (e.g., shame, anger, sadness), while all items related to positive dimensions in both MAS and ASE were above average (considering 6 is the midpoint of the 1–11 scale) for both conditions. This effect is consistent with a previous study [56,57], where no differences were reported on valence (pleasantness, comfort) and arousal dimensions between simulated furnished rooms (cutting edge with rounded furniture and modern with angular edges), albeit those were highly rated when compared to an empty room. One could think that participants reported a pleasant experience in all of the furnished rooms because they were impressed by the degree of realism in those, or by the virtual experience per se. But then the effect would drop after “affective habituation” [82]. As response bias was proposed as a function of presentation order in lengthy sequential preference judgments [83], we controlled for the stimulus presentation sequence and found no main effects. We also did not find any differences in perceived restorativeness, nor in cognitive performance, while Bayes’ factors showed poor evidence of the alternative hypothesis.

In our exploratory analyses of style, results primarily validated our stimuli’s second-level contrast with modern rooms being rated significantly higher than classic ones on the “traditional/novel” scale. This effect was reconfirmed within the Bayesian analysis which indicated strong evidence for the alternative hypothesis. No main difference in the general assessment of style on positive or negative affect or aesthetic value measures was observed, consistent with some previous studies [63,65,66,67], except for complexity and warmth, in favor of classic rooms. While the results of complexity ratings confirmed some previous findings [67], and could be argued as a natural result of the classical style being inclusive of more details (e.g., ornaments) in its principles, the slight difference in the color palette between the two styles could be a confounding factor in the case of warmth. On the other hand, the inclusion of more “green” or “biophilic” features did not impact ratings on naturalness or perceived restorativeness. We also find this surprising as these elements are considered within the biophilic design framework. No other effect of style was found in any of the other outcome variables. Future studies primarily investigating architectural style should aim at maximizing the control for confounding variables by providing well-matched high and low-level properties.

Comparing our almost null results in terms of contour comparison to previous findings of studies that investigated indoor environments and familiar objects, it could be explained by several points. The first concerns the relatively extended viewing time in our study (3 min of exploration time), when compared with previous ones, which mostly relied on gut reactions, by either presenting the stimuli very shortly (84 to 3000 ms) (e.g., [8,31]), or by instructing participants to immediately respond without thinking (e.g., [44]). Previous investigations have found that preference for curved stimuli, which was pronounced under limited times (84 to 150 ms), faded when the stimulus was displayed until response. This finding was replicated with images of real objects [26,35] and abstract shapes [29]. An influence of meaning and semantic content on preference was suggested. In fact, when presenting the same images of indoor environments until response, effects were not consistent with previous studies [4,10,46], and a preference for rectilinear interiors was actually found across the three groups of participants in the most recent study: individuals with autism spectrum condition, neurotypical adults, and design and art students. Another point to consider concerns the use of forced-choice dichotomous scales in some of the previous studies reporting the preference of curved conditions (e.g., beautiful/not beautiful, or like/dislike). The lack of a response options in the middle might have boosted the preference response, as proposed by some researchers, when interpreting the different effects found in their study investigating abstract shapes [29]. We opted for a psychometric 11-point scale to allow for undecided responses. The third point relates to the fact that most of the previous research investigating contour, in general, has targeted similar populations, particularly female participants and psychology students [30], causing limitations in terms of generalizing results. However, we included a rather heterogeneous sample, recruited via more diversified databases. Last but not least, additional potential reasons concern the cultural and individual differences between the populations of the different studies. Culture was proposed to effect aesthetic preference and sensitivity, with the latter suggested change over time, exposure, and perspective [3]. From an architecture point of view, interiors, with the potential affordances (see James Gibson) they create, host the complex interaction between specific atmospheres shaped by different spatial compositions, the perceiver’s characteristics, and their interpretation [5]. A probabilistic model of aesthetic response was proposed to explain the ongoing interaction between humans and their physical environments [50], and identified, in addition to design attributes, a series of factors including biology, personality, social and cultural experience, goals, expectations, associations, and internal constructs. These factors are suggested to contribute to the aesthetic response, impacting affect, physiological response, and behavior. The model acknowledged the complexity of the architectural experience, and further highlighted the major limitation caused by the neglect of the human movement’s influence on the spatial experience in studies that use static stimuli. More than two decades after its publication, most of the known effects still relate to static stimuli rather than real-life experiences.

Even though recent research is targeting inter-individual differences in shape preferences in spaces and objects’ contexts, the role of individual measures on preference is as yet uncertain, requiring further investigations [33,84]. However, when looking closely at previous studies, an interesting sex effect appears. While a curvature preference was observed when the sample predominantly consisted of female psychology, art, and design students [44,49], environments with rectilinear properties were preferred when the sample had a prevalence of male (design students, neurotypical or autistic participants) [49]. The same set of images showed a preference for curved interiors when the sample size consisted of more females than males [8,49]. The authors interpreted the preference for rectilinear spaces in their study as the result of familiarity, which was previously found to be relevant for preference formation [34,38], although other studies investigating drawings of familiar objects have found it to modulate preference for curvature [33]. The sex effect was also found when presenting sketches of familiar objects, where females judged curvilinear objects as more peaceful than males [37]. Additionally, another recent study presenting abstract shapes as stimuli has found that curvature preference was stronger for female students in psychology [30]. This effect was potentially attributed to gender rather than biological determinants. In this present study, post hoc analyses showed a sex effect, however, beyond the preference of one of the conditions over the other (Post-hoc)). Namely, when looking solely at angular rooms, males performed better than females in the cognitive task. Additionally, they rated those rooms higher than females on six out of the 20 affective and spatial dimensions, and reported higher scores on positive affect after exploring them. Although such results could possibly hint at a higher appreciation of angularity in males, this finding is to be interpreted cautiously for many reasons. First, our sample was not balanced in terms of sex and consisted of females more than males. Second, higher scores related to angular conditions do not necessarily indicate the preference of a shape over the other. This suggests that future works could benefit from including equally sized sex groups.

However, there are some limitations to the present study. Although the stimuli were still presented during rating tasks, the evaluation time was relatively long (3 min of exploration vs. an average of 9 min for CT and self-reports). Participants had to provide ratings in each room for 31 questions. This might have caused an effect known as the “museum-fatigue effect” [85], which has been found in many experimental observations and laboratory experiments. Causes were originally attributed to fatigue, but later to other cognitive factors such as satisfaction, information overload, and limitations in attentional capacity [83]. In terms of momentary affect, we had selected a scale that includes a broad range of negative emotions, to evaluate the threat hypothesis previously proposed [31]. However, participants scored very low on negative emotions and used more of the given scale for questions that offered both positive and negative anchors. In future studies, more focus should be directed to positive emotions. Concerning cognitive performance, the selected task was stress-inducing, which is why it is part of the Trier stress test. Although it had proven efficacy in previous studies investigating physical environments [72], it could be that the stress induced by the task might have overlapped the possible effects of contours. 

Future studies may want to focus more strongly on implicit measures of emotions, less stress-inducing cognitive tasks, a lower number of outcome variables, and a more positive set of emotions. Sex could be further explored through the selection of a well-balanced sample. One route is to examine inter-individual differences, which include personality traits and expertise in arts and design. However, more differences should be taken into account, such as cultural background, previous experience with VR, information on familiar and lived architectural environments, among others.

## 5. Conclusions

In summary, while the evidence for curved contour preference in the context of abstract shapes and lines seems robust, it does not appear to be as strong in architectural settings, as multiple studies fail to demonstrate or replicate findings. The present study addressed previous limitations and found that exposure to contrasting contours in virtual interiors within a heterogeneous sample did not elicit significant differences in response to a broad set of psychological dimensions, with tasks and questionnaires administered directly after free exploration, yet within the virtual space, to record an immediate response. The fact that we assessed multiple domains during a close-to-reality architectural experience of fully controlled stimuli, not finding major effects in any of them, makes the study the most comprehensive in the field until now. This suggests that the psychological response to indoor design is much more complex and cannot be reduced into a generalized effect of contour or style, and could involve further multifaceted layers that affect the judgment of spaces on a more individual and contextual level. These results will help to convey a more real-life perspective of the response to the architectural experience in experimental settings and highlight the necessity of further investigations by providing directions for future research.

## Figures and Tables

**Table 1:** Results of the statistical analyses performed on contour conditions using a classical frequentist approach and a Bayesian approach, in addition to the central tendency. Where data is normally distributed, means with standard deviation, and Student *t*-test results are reported. In the case of unmet normality assumption, we report median and IQR, and Wilcoxon signed-rank test results. Effect sizes and alternative hypotheses are also shown for each of the outcome measures.

Dependent Variables	Contour (Angular × Curved)						
	Central Tendency				Classical Frequentist Approach	Bayesian Approach	
	*Angular*		*Curved*		*Paired sample *t*-test*	*Hypothesis*	*BF_01_*
Questionnaire assessing momentary affective state							
*N* = 41; Age = 18–40 (*M* = 27.71); F = 25, M = 16							
Shame	1	(0.5)	1	(0.5)	*Z* = −0.227, *p* = 0.821, *r* = −0.035	angular ≠ curved	BF_01_ = 5.38
Fear	1	(0.5)	1	(0.5)	*Z* = −0.804, *p* = 0.422, *r* = −0.126	angular > curved	BF_01_ = 8.408
Sadness	1	(1)	1	(0.5)	*Z* = 0.267, *p* = 0.789, *r* = 0.042	angular > curved	BF_01_ = 4.75
Happiness	6.99	(±2.38)	7.01	(±2.27)	*t* (40) = −0.128, *p* = 0.899, *d* = 0.02	angular < curved	BF_01_ = 5.356
Anger	1	(0)	1	(0)	*Z* = 1.469, *p* = 0.142, *r* = 0.229	angular > curved	BF_01_ = 1.635
Heartbeat	2	(2)	2	(2)	*Z* = −0.696, *p* = 0.486, *r* = −0.109	angular ≠ curved	BF_01_ = 4.801
Tension	2.5	(2.5)	2.5	(2)	*Z* = 0.193, *p* = 0.847, *r* = 0.03	angular > curved	BF_01_ = 4.855
Activity	8	(3)	8	(3)	*Z* = 1.590, *p* = 0.112, *r* = 0.248	angular ≠ curved	BF_01_ = 1.16
Alertness	8.5	(2)	8.5	(1.5)	*Z* = 0.336, *p* = 0.737, *r* = 0.052	angular ≠ curved	BF_01_ = 5.352
Positivity	9	(2.5)	9	(2)	*Z* = −0.893, *p* = 0.372, *r* = −0.139	angular < curved	BF_01_ = 2.36
Interest	8.5	(2.5)	8.5	(2.5)	*Z* = 1.336, *p* = 0.182, *r* = 0.209	angular ≠ curved	BF_01_ = 2.53
Questionnaire assessing affective and spatial experience							
*N* = 41; Age = 18–40 (*M* = 27.71); F = 25, M = 16							
Peasantness	8.46	(±1.54)	8.82	(±1.39)	*t*(40) = −1.615, *p* = 0.114, *d*= 0.252	angular < curved	BF_01_ = 0.959
Beauty	9	(2.5)	8.5	(1.5)	*Z* = −0.197, *p* = 0.844, *r* = −0.031	angular < curved	BF_01_ = 5.607
**Excitement ^1^**	**7**	**(2.5)**	**7.5**	**(2.5)**	*Z* = −2.009, *p* = 0.046 *, *r* = −0.314	**angular < curved**	**BF_01_ = 0.742**
Spaciousness	7.93	(±1.69)	7.95	(±1.71)	*t*(40) = −0.112, *p* = 0.911, *d* = 0.018	angular ≠ curved	BF_01_ = 5.89
Enclosure	3.91	(±1.59)	3.87	(±1.81 )	*t*(40) = 0.168, *p* = 0.867, *d* = 0.026	angular ≠ curved	BF_01_ = 5.85
Lightness	7	(3)	7	(3)	*Z* = 0.548, *p* = 0.584, *r* = 0.086	angular < curved	BF_01_ = 7.414
Calmness	8.43	(±1.18)	8.50	(±1.41)	*t*(40) = −0.335, *p* = 0.740, *d* = 0.052	angular < curved	BF_01_ = 4.487
Brightness	9.43	(±1.32)	9.35	(±1.6)	*t*(40) = 0.414, *p* = 0.681, *d* = 0.065	angular ≠ curved	BF_01_ = 5.47
Comfort	8	(2)	8.5	(2)	*Z* = −1.617, *p* = 0.106, *r* = −0.252	angular < curved	BF_01_ = 0.774
Cheerfulness	8	(2.5)	8	(1.5)	*Z* = −1.173, *p* = 0.241, *r* = −0.183	angular < curved	BF_01_ = 2.244
Liveliness	7.5	(2.5)	7	(3)	*Z* = −0.026, *p* = 0.980, *r* = −0.004	angular ≠ curved	BF_01_ = 5.641
Familiarity	7.57	(±2.07)	7.23	(±1.98)	*t*(40) = 1.123, *p* = 0.268, *d*= 0.175	angular ≠ curved	BF_01_ = 3.301
**Novelty ^1^**	**7.79**	**(±1.64)**	**6.60**	**(±1.88 )**	*t*(40) = 3.946, *p* < 0.001 ***, *d* = 0.616	**angular ≠ curved**	**BF_01_ = 0.0116**
Simplicity	6.56	(±1.76)	6.13	(±1.86 )	*t*(40) = 1.478, *p* = 0.147, *d* = 0.231	angular > curved	BF_01_ = 1.18
**Order ^1^**	**9.60**	**(±1.05)**	**7.98**	**(±1.76)**	*t*(40) = 6.196, *p* < 0.001 ***, *d* = 0.968	**angular ≠ curved**	**BF_01_ = 0.0000162**
**Harmony ^1^**	**8.45**	**(±1.57)**	**8.98**	**(±1.26)**	*t*(40) = −2.390, *p* = 0.022 *, *d* = 0.373	**angular < curved**	**BF_01_ = 0.241**
Warmth	6.5	(2.5)	7	(3.5)	*Z* = −0.939, *p* = 0.348, *r* = −0.147	angular ≠ curved	BF_01_ = 3.435
Experience	8.40	(±1.51)	8.39	(±1.58)	*t*(40) = 0.053, *p* = 0.958, *d* = 0.008	angular < curved	BF_01_ = 6.172
Naturalness	5.67	(±2.22)	6.12	(±2.26)	*t*(40) = −1.523, *p* = 0.136, *d* = 0.238	angular < curved	BF_01_ = 1.103
**Symmetry ^1^**	**8.26**	**(±1.81)**	**7.66**	**(±1.82)**	*t*(40) = 2.130, *p* = 0.039 *, *d* = 0.333	**angular ≠ curved**	**BF_01_ = 0.779**
Questionnaire on perceived restorativeness							
*N* = 36; Age = 18–40 (*M* = 27.31); F = 23, M = 13							
Total score	3.10	(±0.54 )	3.16	(±0.52)	*t*(35) = −0.789, *p* = 0.436, *d* = 0.131	angular < curved	BF_01_ = 2.7
					Cognitive task scores		
*N* = 42; Age = 18–40 (*M* = 27.55); F = 27, M = 15							
CT scores	9.86	(8.56)	9.56	(9.25)	*Z* = −0.431, *p* = 0.666, *r* = −0.067	angular < curved	BF_01_ = 5.123

^1^ Rows in bold indicate statistically significant outcome measures (bolded for ease of reference). Significance is also marked with asterisks next to *p*-values.

**Table 2:** Results of the statistical analyses performed on style conditions using a classical frequentist approach and a Bayesian approach, in addition to the central tendency. Where data are normally distributed, means with standard deviation, and Student *t*-test results are reported. In the case of unmet normality assumption, we report median and IQR, and Wilcoxon signed-rank test results. Effect sizes and alternative hypotheses are also shown for each of the outcome measures.

Dependent Variables	Style (Modern × Classic)						
	Central Tendency				Classical Frequentist Approach	Bayesian Approach	
	*Modern*		*Classic*		*Paired sample *t*-test*	*Hypothesis*	*BF_01_*
Questionnaire assessing momentary affective state							
*N* = 41; Age = 18–40 (*M* = 27.71); F = 25, M = 16							
Shame	1	(0.5)	1	(0.5)	*Z* = −0.261, *p* = 0.794 *r* = −0.041	modern ≠ classic	BF_01_ = 5.171
Fear	1	(0.5)	1	(0.5)	*Z* = −1.103, *p* = 0.27, *r* = −0.172	modern ≠ classic	BF_01_ = 4.069
Sadness	1	(1)	1	(0.5)	*Z* = 0.118, *p* = 0.906, *r* = 0.018	modern ≠ classic	BF_01_ = 5.08
Happiness	7.5	(3)	7.5	(3.5)	*Z* = −0.823, *p* = 0.411 *r* = −0.128	modern ≠ classic	BF_01_ = 4.265
Anger	1	(0.5)	1	(0)	*Z* = 1.718, *p* = 0.086, *r* = 0.268	modern ≠ classic	BF_01_ = 2.15
Heartbeat	2	(2)	2	(2)	*Z* = −1.464, *p* = 0.143, *r* = −0.229	modern ≠ classic	BF_01_ = 2.003
Tension	3.39	(±2.01)	3.29	(±1.97)	*t*(40) = 0.555, *p* = 0.582, *d* = 0.087	modern ≠ classic	BF_01_ = 5.129
Activity	8	(2)	8.5	(2.5)	*Z* = 0.508, *p* = 0.612, *r* = 0.079	modern ≠ classic	BF_01_ = 4.701
Alertness	8.38	(±1.61)	8.30	(±1.86)	*t*(40) = 0.458, *p* = 0.650, *d* = 0.072	modern ≠ classic	BF_01_ = 5.37
Positivity	9	(2)	9	(2.5)	*Z* = −1.151, *p* = 0.250, *r* = −0.180	modern ≠ classic	BF_01_ = 4.462
Interest	8.5	(1.5)	8.5	(2)	*Z* = 0.690, *p* = 0.49, *r* = 0.109	modern ≠ classic	BF_01_ = 4.35
Questionnaire assessing affective and spatial experience							
*N* = 41; Age = 18–40 (*M* = 27.71); F = 25, M = 16							
Pleasantness	9	(1.5)	9	(1)	*Z* = −0.210, *p* = 0.834, *r* = −0.033	modern ≠ classic	BF_01_ = 5.608
Beauty	8.5	(1.5)	9	(2)	*Z* = −0.937, *p* = 0.349 *r* = −0.146	modern ≠ classic	BF_01_ = 4.36
Excitement	7.07	(±1.84)	7.17	(±1.96)	*t*(40) = −0.315, *p* = 0.754, *d* = 0.049	modern ≠ classic	BF_01_ = 5.657
**Spaciousness** **^1^**	**8.5**	**(2)**	**7.5**	**(3)**	***Z*** = 2.487, *p* = 0.0129 *, *r* = 0.388	**modern ≠ classic**	**BF_01_ = 0.139**
**Enclosure** **^1^**	**3.5**	**(1.5)**	**4**	**(2)**	***Z*** = −2.452, *p* = 0.014 *, *r* = −0.383	**modern ≠ classic**	**BF_01_ = 0.275**
Lightness	7.21	(±1.92)	7.01	(±1.86)	*t*(40)= 0.750, *p* = 0.458, *d* = 0.117	modern ≠ classic	BF_01_ = 4.55
Calmness	8.61	(±1.23)	8.32	(±1.62)	*t*(40) = 0.998 *p* = 0.324, *d* = 0.156	modern ≠ classic	BF_01_ = 3.726
Brightness	9.5	(1.5)	10	(2.5)	*Z* = 1.617, *p* = 0.106, *r* = 0.253	modern ≠ classic	BF_01_ = 1.068
Comfort	8.07	(±1.78)	8.05	(±2.06)	*t*(40) = 0.060, *p* = 0.952, *d* = 0.009	modern ≠ classic	BF_01_ = 5.918
Cheerfulness	7.93	(±1.51)	7.99	(±1.69)	*t*(40) = −0.213, *p* = 0.832, *d* = 0.033	modern ≠ classic	BF_01_ = 5.803
Liveliness	6.98	(±2.16)	7.39	(±2.02)	*t*(40) = −1.121, *p* = 0.269, *d* = 0.175	modern ≠ classic	BF_01_ = 3.307
Familiarity	7.5	(3)	7.5	(3.5)	*Z* = 1.113, *p* = 0.266, *r* = 0.174	modern ≠ classic	BF_01_ = 4.528
**Novelty** **^1^**	**9**	**(1.5)**	**4.5**	**(3)**	***Z*** = 5.308, *p* < 0.001 ***, *r* = 0.829	**modern > classic**	**BF_01_ = 0.0000576**
**Simplicity** **^1^**	**7.34**	**(±1.79)**	**5.35**	**(±1.93)**	***t*** (40) = 6.262, *p* < 0.001 ***, *d* = 0.978	**modern ≠ classic**	**BF_01_ = 0.00001322**
**Order** **^1^**	**9.70**	**(±1.09)**	**9.15**	**(±1.42)**	***t*** (40) = 2.780, *p* = 0.008 **, *d* = 0.434	**modern ≠ classic**	**BF_01_ = 0.21**
Harmony	8.78	(±1.46)	8.65	(±1.64 )	*t*(40) = 0.458, *p* = 0.649, *d* = 0.072	modern ≠ classic	BF_01_ = 5.371
**Warmth** **^1^**	**6.26**	**(±2.27)**	**7.39**	**(±1.98)**	***t*** (40) = −3.236, *p* = 0.002 **, *d* = 0.505	**modern ≠ classic**	**BF_01_ = 0.0727**
Experience	8.39	(±1.56)	8.40	(±1.73)	*t*(40) = −0.042, *p* = 0.967, *d* = 0.007	modern ≠ classic	BF_01_ =5.924
Naturalness	5.65	(±2.28)	6.15	(±2.37)	*t*(40) = −1.411, *p* = 0.166, *d* = 0.220	modern ≠ classic	BF_01_ = 2.37
Symmetry	8.06	(±1.83)	7.85	(±1.74 )	*t*(40) = 0.793, *p* = 0.432, *d* = 0.124	modern ≠ classic	BF_01_ = 4.416
Questionnaire on perceived restorativeness							
*N* = 36; Age = 18–40 (*M* = 27.31); F = 23, M = 13							
Total score	3.07	(±0.6)	3.20	(±0.67)	*t*(35) = −0.942, *p* = 0.352, *d* = 0.157	modern ≠ classic	BF_01_ = 3.7
					Cognitive task scores		
*N* = 42; Age = 18–40 (*M* = 27.55); F = 27, M = 15							
CT scores	10.58	(10.14)	9.50	(7.61)	*Z* = 0.594, *p* = 0.553, *r* = 0.092	modern ≠ classic	BF_01_ = 4.791

^1^ Rows in bold indicate statistically significant outcome measures (bolded for ease of reference). Significance is also marked with asterisks next to *p*-values.

## Data Availability

Data is available from the corresponding authors upon request.

## Supplementary Materials

The following are available online at https://www.mdpi.com/article/10.3390/ijerph182312510/s1, Table S1: Affective and spatial experience (ASE) dimensions with their respective domains, along with the tagged descriptive adjectives and numeric scales, Table S2: Momentary affective state (MAS) dimensions with their respective domains, along with the tagged descriptive adjectives and numeric scales, Table S3: Perceived Restorativeness Scale (PRS) items, along with the respective original subscales they represent, Table S4: Included/excluded participants for each set of measures, along with reasons for exclusion, Table S5: Scores on the SSQ, including the three subscales (Nausea, Oculomotor disturbance, and Disorientation), in addition to the total score, Post-hoc: Exploratory analysis of sex, Videos: Short videos inside the rooms
