# Supplementary material for: The Living Space: Psychological Well-Being and Mental Health in Response to Interiors Presented in Virtual Reality
Source: Int J Environ Res Public Health. 2021 Nov 27;18(23):12510. doi: 10.3390/ijerph182312510 (PMC8656816; doi:10.3390/ijerph182312510)
Supplement: Supplementary file 1 [file ijerph-18-12510-s001.zip › ijerph-1407869-supplementary.pdf]

International Journal of
Environmental Research and Public Health

MDPI

Supplementary Materials

1. Tables and Figures

Table S1. Affective and spatial experience (ASE) dimensions with their respective domains, along with the tagged descriptive adjectives and numeric scales.

Dimension	Anchors	Left	Right
Pleasantness	unpleasant vs. pleasant	5 = describes strongly	5 = describes strongly
Beauty	ugly vs. beautiful	5 = describes strongly	5 = describes strongly
Excitement	not exciting vs. exciting	5 = describes strongly	5 = describes strongly
Spaciousness	narrow vs. spacious	5 = describes strongly	5 = describes strongly
Enclosure	open vs. closed	5 = describes strongly	5 = describes strongly
Lightness	overwhelming vs. light	5 = describes strongly	5 = describes strongly
Calmness	stressful vs. calming	5 = describes strongly	5 = describes strongly
Brightness	dark vs. bright	5 = describes strongly	5 = describes strongly
Comfort	uncomfortable vs. comfortable	5 = describes strongly	5 = describes strongly
Cheerfulness	depressing vs. cheerful	5 = describes strongly	5 = describes strongly
Liveliness	lifeless vs. lively	5 = describes strongly	5 = describes strongly
Familiarity	unfamiliar vs. familiar	5 = describes strongly	5 = describes strongly
Novelty	traditional vs. novel	5 = describes strongly	5 = describes strongly
Simplicity	complex vs. simple	5 = describes strongly	5 = describes strongly
Order	chaotic vs. ordered	5 = describes strongly	5 = describes strongly
Harmony	not harmonious vs. harmonious	5 = describes strongly	5 = describes strongly
Warmth	cold vs. warm	5 = describes strongly	5 = describes strongly
Experience	bad vs. good	5 = describes strongly	5 = describes strongly
Naturalness	artificial vs. natural	5 = describes strongly	5 = describes strongly
Symmetry	asymmetrical vs. symmetrical	5 = describes strongly	5 = describes strongly

Table S2. Momentary affective state (MAS) dimensions with their respective domains, along with the tagged descriptive adjectives and numeric scales.

Domain	Dimension	Anchors	Left	Right
Emotional feeling	Shame	embarrassed / ridiculed / ashamed / foolish	0 = little	10 = too much
	Fear	frightened / timid / afraid / scared	0 = little	10 = too much
	Sadness	sad / depressed / miserable / dejected	0 = little	10 = too much
	Happiness	happy / gay / cheerful / delighted	0 = little	10 = too much
	Anger	angry / annoyed / mad / sore	0 = little	10 = too much
Bodily sensation	Heartbeat	sensation of a pounding heart	0 = little	10 = too much
Arousal & valence	Relaxation vs. tension	(calm / relaxed / placid / at ease) vs. (nervous / restless / tense / wound up)	5 = applies very much	5 = applies very much
	Tiredness vs. activity	(tired / fatigued / sluggish / exhausted) vs. (energetic / active / animated / lively)	5 = applies very much	5 = applies very much
	Negativity vs. positivity	(negative / unpleasant) vs. (positive / pleasant)	5 = applies very much	5 = applies very much
Cognitive states	Confusion vs. alertness	(confused /baffled / perplexed) vs. (alert / attentive / receptive / lucid)	5 = applies very much	5 = applies very much
Motivational states	Boredom vs. interest	(bored / indifferent / dull) vs. (curious / interested / motivated)	5 = applies very much	5 = applies very much

Table S3. Perceived Restorativeness Scale (PRS) items, along with the respective original subscales they represent: BA=Being away, COM=Compatibility, COH=Coherence, FA=Fascination, SCO=Scope.

Statement	Original subscale	Top	Bottom
This place piques my curiosity.	FA	0 = not true at all	4 = absolutely true
There are a lot to explore and discover here.	FA	0 = not true at all	4 = absolutely true
A lot of interesting things get my attention here.	FA	0 = not true at all	4 = absolutely true
The things and processes that I observed here are in natural harmony.	COH	0 = not true at all	4 = absolutely true
This place is fascinating.	FA	0 = not true at all	4 = absolutely true
To be here corresponds to my personal inclinations.	COM	0 = not true at all	4 = absolutely true
It is easy to do what I want here.	COM	0 = not true at all	4 = absolutely true
This place is a world of its own.	SCO	0 = not true at all	4 = absolutely true
Everything seems to have its place here.	COH	0 = not true at all	4 = absolutely true
When I stay here, nothing unwanted disturbs my concentration.	BA	0 = not true at all	4 = absolutely true
Spending time here creates a welcome change from my daily routine.	BA	0 = not true at all	4 = absolutely true
In this place the order of things is easy to see.	COH	0 = not true at all	4 = absolutely true

Table S4. Included/excluded participants for each set of measures, along with reasons for exclusion.

Measure	Excluded	Reason	N
General	3	dropped out (simulation sickness symptoms or headache)	45
ASE & MAS	4	technical errors[1]	41
CP	3	technical errors [1]	42
PRS	9	missing values[2]	36

[1] Technical errors include software/hardware errors, which resulted in either drop out before the end of the experiment, or multiple restarts leading to missing values in the datasets. [2] Missing values could have been due to a question not answered, or errors in data extraction.

Table S5. Scores on the SSQ, including the three subscales (Nausea, Oculomotor disturbance, and Disorientation), in addition to the total score.

Measure	Mdn	IQR	Range	Mdn	IQR	Range
		pre			post	
Nausea (N)	9.54	19.08	(0-19.08)	9.54	28.62	(0-28.62)
Oculomotor Disturbance (O)	7.58	15.16	(0-15.16)	22.74	22.27	(15.16-37.90)
Disorientation (D)	0.00	13.92	(0-13.92)	13.92	41.76	(0-41.76)
**Total SS score (TS)**	**7.48**	**22.44**	**(0-22.44)**	**26.18**	**29.92**	**(11.22-41.14)**

2. Exploratory Analysis of Sex (Post-Hoc)

As we attempted to control for sex, post-hoc analyses revealed a specific effect of both angular and modern rooms when comparing males and females' responses in each of the conditions separately. This effect was present in 3 out of 4 sets of outcome measures in the contour comparison, and 2 sets when comparing style.

Within the contour analysis, males rated angular rooms higher than females on 6 dimensions of the ASE out of the 20 tested: calmness ($t(36.423) = 2.905$, $p = 0.006$), cheerfulness ($t(38.356) = 2.803$, $p = 0.008$), excitement ($t(35.862) = 2.149$, $p = 0.034$), liveliness ($t(36.562) = 2.588$, $p = 0.0138$), familiarity ($t(38.606) = 2.525$, $p = 0.0158$), and experience ($t(38.195) = 2.118$, $p = 0.047$). Consistently, they reported higher on the positive emotions of the MAS, where they felt significantly more interested ($t(38.971) = 2.156$, $p = 0.034$), happy ($t(39) = 2.52$, $p = 0.016$), positive ($t(36.692) = 2.081$, $p = 0.044$), and active ($t(38.813) = 2.286$, $p = 0.028$) than women after angular rooms. They also performed better than females on the cognitive task after exploring angular rooms ($t(20.601) = 2.197$, $p = 0.039$).

The sex effect extended to room style, within the same 4 above mentioned positive dimensions of the MAS, where males reported on their momentary affective state with significantly higher values than females, after being exposed to modern rooms. Males reported more interest ($t(38.58) = 2.97$, $p = 0.005$), alertness ($t(37.53) = 2.75$, $p = 0.009$), happiness ($t(38.99) = 2.47$, $p = 0.018$), positivity ($t(31.27) = 2.127$, $p = 0.04$), and activity ($t(38.89) = 3.03$, $p = 0.004$). Moreover, they rated modern rooms higher than women on 7 out of the ASE's 20 dimensions: calmness ($t(37.87) = 2.072$, $p = 0.045$), cheerfulness ($t(37.01) = 2.944$, $p = 0.005$), excitement ($t(36.774) = 2.472$, $p = 0.018$), liveliness ($t(31.428) = 2.072$, $p = 0.009$), familiarity ($t(37.235) = 3.455$, $p = 0.001$), pleasantness ($t(38.722) = 2.06$, $p = 0.045$), and comfort ($t(38.424) = 2.99$, $p = 0.005$).
